# The effect of thickness and sintering mode on the impact strength of a graded Zirconia

**DOI:** 10.4317/jced.64108

**Published:** 2026-04-25

**Authors:** Beatriz Serralheiro da Cruz, Amanda M. de Oliveira Dal Piva, João Paulo Mendes Tribst, Isabella Marian Lena, Arie Werner, Renata Marques de Melo, Cornelis Johannes Kleverlaan

**Affiliations:** 1 Department of Dental Materials and Prosthodontics, Institute of Science and Technology, Sao Paulo State University (UNESP), São José dos Campos, SP, Brazil; 2 Department of Dental Materials, Academic Centre for Dentistry Amsterdam (ACTA), Universiteit van Amsterdam and Vrije Universiteit, Amsterdam, Netherlands; 3 Department of Reconstructive Oral Care, Academic Centre for Dentistry Amsterdam (ACTA), Universiteit van Amsterdam and Vrije Universiteit, Amsterdam, Netherlands; 4 Post-Graduate Program in Oral Science, Faculty of Odontology, Federal University of Santa Maria (UFSM), Santa Maria, RS, Brazil

## Abstract

**Background:**

Graded zirconia ceramics balance the high strength of 3Y-TZP with the translucency of 5Y-PSZ through their layered microstructure. However, the mechanical behavior of distinct compositional zones, particularly the transition zone, remains insufficiently characterized under dynamic loading conditions. This study aimed to evaluate the influence of different sintering protocols and thicknesses on the impact strength of graded zirconia.

**Material and Methods:**

Specimens were sectioned by layer (3Y-TZP, transition zone, 5Y-PSZ) under two thicknesses (1.0 and 1.5 mm) and two sintering protocols (conventional and speed). Impact strength testing, finite element analysis, and fractographic analysis were performed. A total of 180 bars were tested using the Dynstat method, with results analyzed via three-way ANOVA ( = 0.05).

**Results:**

The 3Y-TZP layer exhibited the highest impact strength (46.8-66.7 kJ/m²), followed by the transition zone (23.7-41.9 kJ/m²) and 5Y-PSZ (15.5-28.7 kJ/m²). A significant three-way interaction (F2,168 = 5.080, p = 0.007) was observed, with thicker 5Y-PSZ specimens showing ~45% less resistance under speed sintering. Fractographic analysis revealed intergranular fracture without delamination, confirming structural integrity.

**Conclusions:**

The 3Y-TZP layer provides superior impact resistance regardless of processing conditions, while conventional sintering is preferable for thicker 5Y-PSZ layers. These findings inform material selection and manufacturing processes for graded zirconia restorations in high-stress applications.

## Introduction

Dental ceramics have gained widespread acceptance in prosthetic rehabilitation due to their favorable optical and biomechanical properties, as well as the ease of fabrication using CAD/CAM systems (1). Among these materials, zirconia-based ceramics have garnered significant attention due to their superior mechanical strength and transformation toughening mechanism, which stems from zirconium oxide's polymorphic nature existing in monoclinic, tetragonal, and cubic crystallographic phases. The stress-induced transformation from tetragonal to monoclinic phase generates a volume increase of approximately 4-5%, creating crack-tip shielding that substantially enhances fracture resistance (2). However, while tetragonal grains provide a beneficial toughening mechanism, cubic grains offer superior light transmission, which is essential for achieving highly aesthetic restorations, although with decreased brittleness (3). The pursuit of combining these complementary properties has driven significant material science innovations using yttria (Y2O3) as the predominant stabilizer, creating yttria-stabilized zirconias (YSZ) with tailored characteristics: 3Y-TZP (3 mol% yttria) exhibits a predominantly tetragonal microstructure with high strength (0.9-1.2 GPa) but limited translucency, while 5Y-PSZ (5 mol% yttria) incorporates substantial cubic phase content, reducing strength (0.4-0.7 GPa) while dramatically improving translucency (4). To address the inherent trade-off between mechanical strength and optical properties, manufacturers have developed innovative multilayered approaches that strategically combine different zirconia compositions within a single restoration (5). These sophisticated multilayer systems feature polychromatic designs that mimic natural tooth translucency gradation through compositionally graded structures. A particularly innovative approach involves structurally graded zirconia, which incorporates distinct microstructural zones within a single disc through Gradient Technology (GT), creating smooth compositional transitions with higher yttria content in the enamel layer and lower content in the dentin layer to optimize aesthetic and mechanical properties (5 - 8). This technology potentially eliminates stress concentration points and increases fatigue resistance and interfacial toughness (9) while maintaining clinical aesthetics. ZirCAD Prime® (Ivoclar, Schaan, Liechtenstein) exemplifies this advancement, featuring a vertical gradient from a predominantly cubic enamel layer (5Y-PSZ) to a tetragonal dentin/core layer (3Y-TZP), with a transition zone (TZ) connecting these distinct regions. This design aims to optimize both aesthetic outcomes and mechanical resistance by providing zone-specific properties within a monolithic structure. However, despite the clinical innovation of graded zirconia systems, this intermediate transition zone where microstructural phases coexist and compositional gradients occur requires further investigation, as it represents a complex interaction zone that could lead to potential mechanical vulnerability . The coexistence of different crystallographic phases may generate microstructural heterogeneities, residual stress concentrations, or mismatched coefficients of thermal expansion (CTE), potentially compromising long-term mechanical integrity (10 , 11). This heterogeneity is particularly critical since different zones within graded zirconia exhibit distinct responses to factors such as aging, impacting long-term properties (6). Recent studies confirm that the transition zone exhibits mechanical properties, including microhardness, elastic modulus, and fracture toughness, with values intermediate between the dentin and enamel zones, validating the gradient property concept (5 , 8). Understanding the mechanical behavior of this critical interface under varying loading conditions becomes essential for predicting clinical performance, particularly as sintering protocols significantly influence the microstructural development and phase distribution within these complex multilayer systems. Sintering protocols influence zirconia's final microstructure, affecting grain size, phase distribution, and porosity through manipulation of temperature and heating or cooling rates (12 - 22). While manufacturers have introduced accelerated sintering protocols (speed sintering) to improve clinical efficiency and reduce processing time, their effects on structurally graded materials remain under investigation. High heating rates may induce transient residual stresses and alter phase distribution (16 , 22), particularly in cubic-rich regions that show increased susceptibility to thermal damage (22). The literature lacks consensus on how different sintering approaches impact the mechanical properties of graded zirconia. Lubauer et al. reported that high-speed sintering tends to cause a significant decrease in flexural strength primarily in 5Y-PSZ zirconia, while zirconias with lower yttria content (3Y- and 4Y-PSZ) showed minimal or no significant changes, likely due to differences in microstructural susceptibility to thermal effects (19).The scarcity of comprehensive data on the effects of these protocols on graded zirconia highlights the need for further research to fully assess their implications for long-term clinical performance under varying loading conditions. From a clinical perspective, restorative materials must withstand sudden, high-magnitude forces encountered during masticatory trauma, bruxism episodes, or accidental occlusal contacts. While the literature encompasses a variety of mechanical tests, including flexural, tensile, and compressive evaluations that are widely utilized to assess restorative material properties, traditional static testing methods fail to capture the dynamic nature of these clinical scenarios. Impact strength testing using standardized methodologies provides enhanced simulation of clinical loading scenarios by applying high-strain-rate loads that more closely approximate sudden clinical forces. This approach reveals distinct fracture patterns and enables more accurate characterization of material toughness under abrupt stress conditions, information that quasi-static tests cannot adequately provide (23 , 24). In graded zirconia systems, strain rate significantly influences crack propagation behavior: while 3Y-TZP regions benefit from transformation toughening mechanisms that effectively limit crack tip velocity, cubic-rich zones may exhibit fundamentally different failure responses under identical dynamic loading conditions. This microstructural heterogeneity necessitates comprehensive evaluation of graded zirconia systems under impact loading to understand their clinical performance potential. Given the heterogeneous microstructure of graded zirconias and the documented mechanical differences between their constituent layers, evaluating impact strength under clinically relevant conditions becomes crucial. Specimen thickness may directly influence energy absorption capacity and fracture characteristics (25), while sintering protocols may affect phase stability and residual stress distribution throughout the graded structure. Previous investigations have documented flexural strength variations across ZirCAD Prime® layers, with recent morphological and mechanical studies consistently demonstrating that the dentin zone exhibits the highest resistance, while the transition zone shows intermediate values (26 , 27). However, no studies have assessed the impact strength of these distinct zones, particularly considering variable thicknesses and sintering protocols. This knowledge gap represents a significant limitation in understanding graded zirconia clinical performance, particularly given the increasing adoption of these materials in high-stress applications. Comprehensive characterization of impact strength across different zones, thicknesses, and processing conditions is essential for evidence-based clinical decision-making and optimal restoration design. Therefore, this study aims to evaluate the impact strength of the three microstructural zones (enamel, transition, and body) of a graded zirconia, under two different specimen thicknesses and sintering protocols. The null hypothesis is that there would be no significant differences in impact strength between zones, thicknesses, or sintering modes.

## Material and Methods

**Figure 1:**
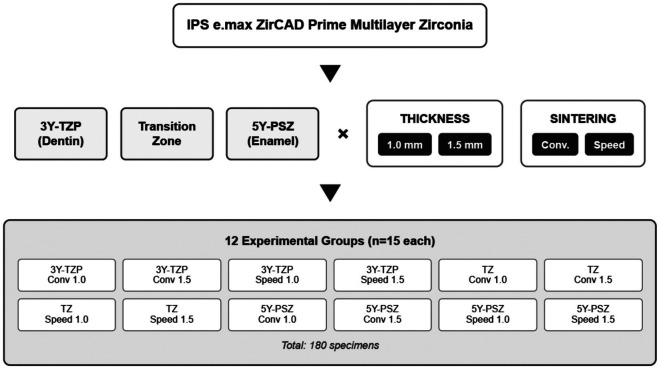
Study design.

The selected material for this study was IPS e.max ZirCAD Prime graded zirconia (Ivoclar, Schaan, Liechtenstein), which, according to the manufacturer's specifications, features a 3 mm 5Y-PSZ incisal zone, a 4 mm transition zone (TZ), and a 3Y-TZP dentin zone ranging from 9 to 18 mm. Samples were obtained from all three zones of the disc: 5Y-PSZ, TZ, and 3Y-TZP. Sample thickness varied between 1.0 and 1.5 mm, simulating clinical restoration thickness, and both conventional and speed sintering modes were evaluated. The study design and group distribution are presented in Figure 1.

1

- Sample preparation Zirconia pre-sintered bars (N=180) were extracted from the center of each respective layer according to manufacturer specifications using a precision cutting machine (Isomet® 1000, Precision Sectioning Saw, Buehler, Lake Bluff, Illinois, USA) with a diamond saw (Series 15LC Diamond Blade wafering, Buehler, USA) under constant water cooling. Subsequently, manual grinding procedures were performed on the samples under water using sequential #240, #400, and #600 grits until the final dimensions of 19 × 5 × 1.25 or 1.9 mm were achieved, considering a shrinkage rate of approximately 20% after sintering. Afterward, the specimens were sintered according to the manufacturer's protocol. The speed sintering (2h26min) was performed using a Programat® S2 Sintering Furnace (Ivoclar, Schaan, Liechtenstein), while the conventional sintering (9h50min) was performed using a Programat® S1 1600 Sintering Furnace (Ivoclar, Schaan, Liechtenstein). Both furnaces were set to the closed sintering programs for the chosen material, according to the group's distribution, strictly following the manufacturer's sintering setup. The final measurement of the specimens was taken with a digital caliper to ensure the dimensions of 15 × 4 × 1.0 or 1.5 mm for the impact strength testing. - Impact Strength Test The impact strength assessment was performed using the Dynstat method, which consists of the dynamic breaking of the sample and reading the amount of energy used to break it on the equipment's scale. A Dynstat apparatus (Zwick &amp; Co., Eisingen, Germany) was used according to ISO 13802, in which an impact test was performed by dropping a pendulum to strike the sample fixed in a jig. The test was conducted with a pendulum energy of 5 kpcm (~0.5 J) at room temperature. The specimens were tested dry, assuming that ceramic pieces do not absorb water in clinical situations. The corrected absorbed energy (Ec) was recorded, and the impact strength (adU) of each specimen was calculated in kJ/m² using Equation 1 (23 , 24). 


$$a_{dU}=\frac{E_{c}}{wB}\times 10^{3}$$

Where E_c is the corrected energy absorbed by breaking the specimen (J), w is the width and B is the thickness of the specimen (mm). After the impact test, the fractured specimens were analyzed using a stereomicroscope (Discovery V20, Carl Zeiss, Jena, Thuringia, Germany) at 50× magnification. Representative specimens were selected, ultrasonically cleaned in isopropyl alcohol for 480 seconds, dried with an air spray and in an oven (37°C) overnight, gold-sputtered, and selected for scanning electron microscopy (SEM; Evo LS15, Carl Zeiss, Göttingen, Germany) to map the fracture direction and origin, as well as fractographic marks. - Finite Element Analysis (FEA) A numerical simulation using three-dimensional (3D) FEA was applied to calculate the stress concentration at different thicknesses under the impact strength test conditions. The test setup was designed in modeling software (Rhinoceros version 5.0 SR8, 2013, McNeel North America, Seattle, WA, USA) to replicate the in vitro condition for both evaluated thicknesses. The volumetric solids were exported to computer-aided engineering software (ANSYS 19.2, ANSYS Inc., Houston, TX, USA) and replicated according to the evaluated thicknesses. The mesh convergence test was performed to define a mesh composed of 51,924 nodes and 12,847 tetrahedral elements for the model with 1.0 mm thickness, and 52,715 nodes and 13,348 tetrahedral elements for the model with 1.5 mm thickness. The Young's modulus of the tested zirconia was set at 200 GPa, and the Poisson's ratio at 0.3 (4), considering an isotropic and linear behavior. The contacts were considered frictional. Similar to Jäger et al. (2021) (24), energy losses were ignored in this study, and the impact on the specimen was represented only as a static force causing a displacement resulting in displacement energy. In order to calculate the inputs considered for the impact test setup, the following Equation 2 was used: 


$$\theta^{\prime \prime }=\frac{g}{R}\,\sin\theta$$

Where '' is the angular acceleration, g is the acceleration due to gravity (approximately 9.81 m/s2), R is the length of the pendulum, and is the angle of the pendulum. Substituting the given values into the Equation 3,4,5: Therefore, the angular acceleration of the pendulum is 28.72 rad/s2.


$$\theta^{\prime \prime }=-\frac{9.81\;\frac{m}{s^{2}}}{0.25\;m}\times \sin\;47.15{}^{\circ}$$

$$\theta^{\prime \prime }=-39.24\;\frac{\mathrm{rad}}{s^{2}}\times 0.7316$$

$$\theta^{\prime \prime }=-28.72\;\frac{\mathrm{rad}}{s^{2}}$$

Next, the force acting on a pendulum can be calculated. Since the pendulum rotates around a fixed point, Newton's second law of motion can be used to calculate the force using the angular acceleration of the pendulum (). It states that force (F) is equal to the mass (m) of an object multiplied by its acceleration (a). In this case, the acceleration corresponds to the angular acceleration (). The formula is given in Equation 6:


$$F=m\times \theta^{\prime \prime }$$

Substituting the given values, the force acting on the pendulum was approximately 5.02 N. After the meshing process, the model was fixed in the test device, and a standardized abrupt load was applied, simulating the same in vitro impact. The maximum principal stress (MPa) was measured. - Statistical analysis Data were analyzed using Jamovi software (version 2.6). Statistical significance was set at = 0.05. All analyses were performed with equal sample sizes (n = 15 per group). A three-way factorial design was used to evaluate the main effects of zirconia type (3Y-TZP, TZ, 5Y-PSZ), sintering protocol (conventional, speed), and thickness (1.0 mm, 1.5 mm), as well as their two-way and three-way interactions on impact strength. When significant interactions were detected, post hoc comparisons were conducted using Games-Howell tests to account for variance heterogeneity.

## Results

- Impact strength test Impact strength results were recorded for all groups. Descriptive statistics (mean ± standard deviation) are presented in Table 1.

Table 1

**Figure 2:**
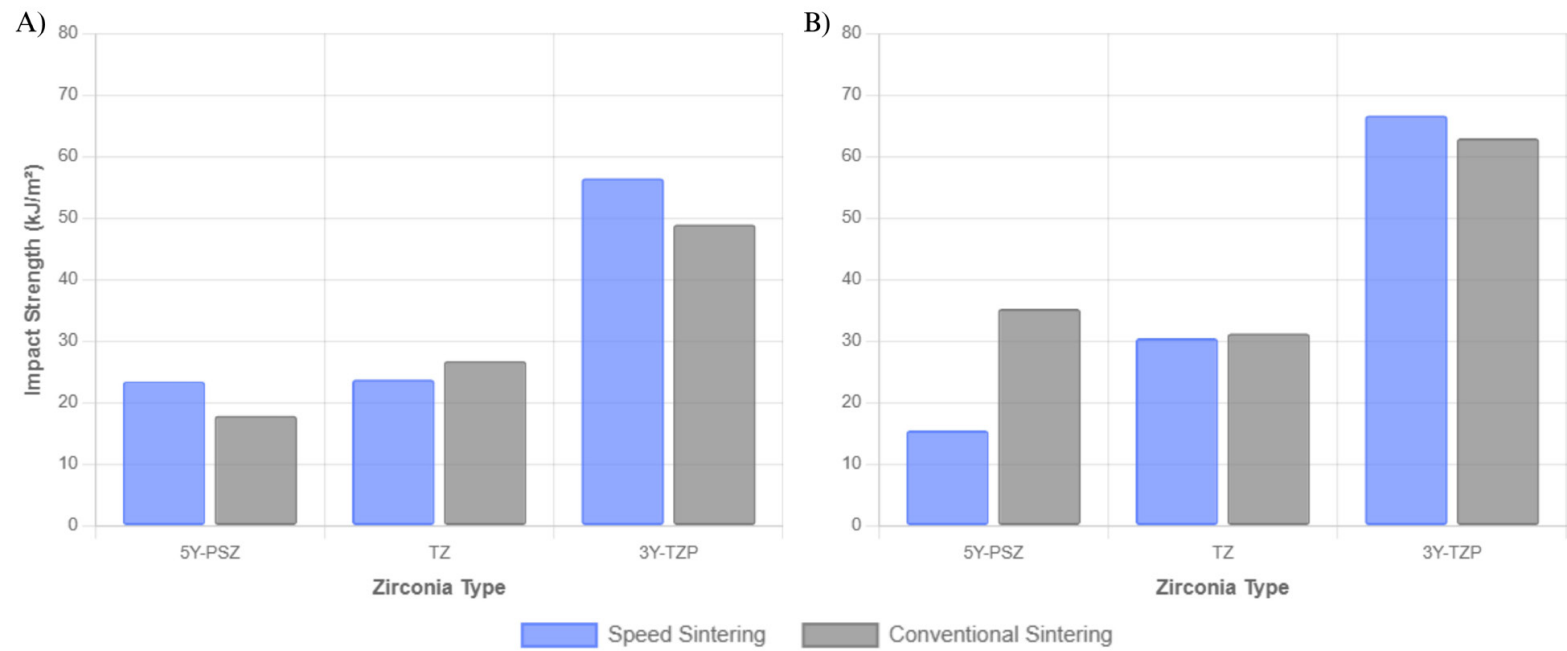
Mean impact strength (kJ/m²) of different zirconia types under conventional and speed sintering protocols. (A) Results for 1.0 mm specimens. (B) Results for 1.5 mm specimens.

The lowest values were observed in the 5Y-PSZ group (15.5 ± 3.31 kJ/m²), while the highest values were found in the 3Y-TZP group (66.7 ± 23.1 kJ/m²), both under speed sintering conditions at 1.5 mm thickness, highlighting the distinct mechanical behavior of each layer within the graded zirconia system (Fig. 2).

2

The Shapiro-Wilk test indicated normality violations in some groups, while Levene's test revealed significant heterogeneity of variances across multiple comparisons (p &lt; 0.05), justifying the use of Welch's ANOVA approach. The three-way factorial ANOVA revealed significant main effects for zirconia type and thickness, while sintering protocol showed no significant main effect. A significant three-way interaction among zirconia type, sintering protocol, and thickness was detected (F2,168 = 5.080, p = 0.007), indicating that the optimal combination of parameters cannot be determined by examining main effects alone. Significant two-way interactions were also observed for zirconia × sintering and sintering × thickness, further supporting the complex interdependence among factors (Table 2).

Table 2

**Figure 3:**
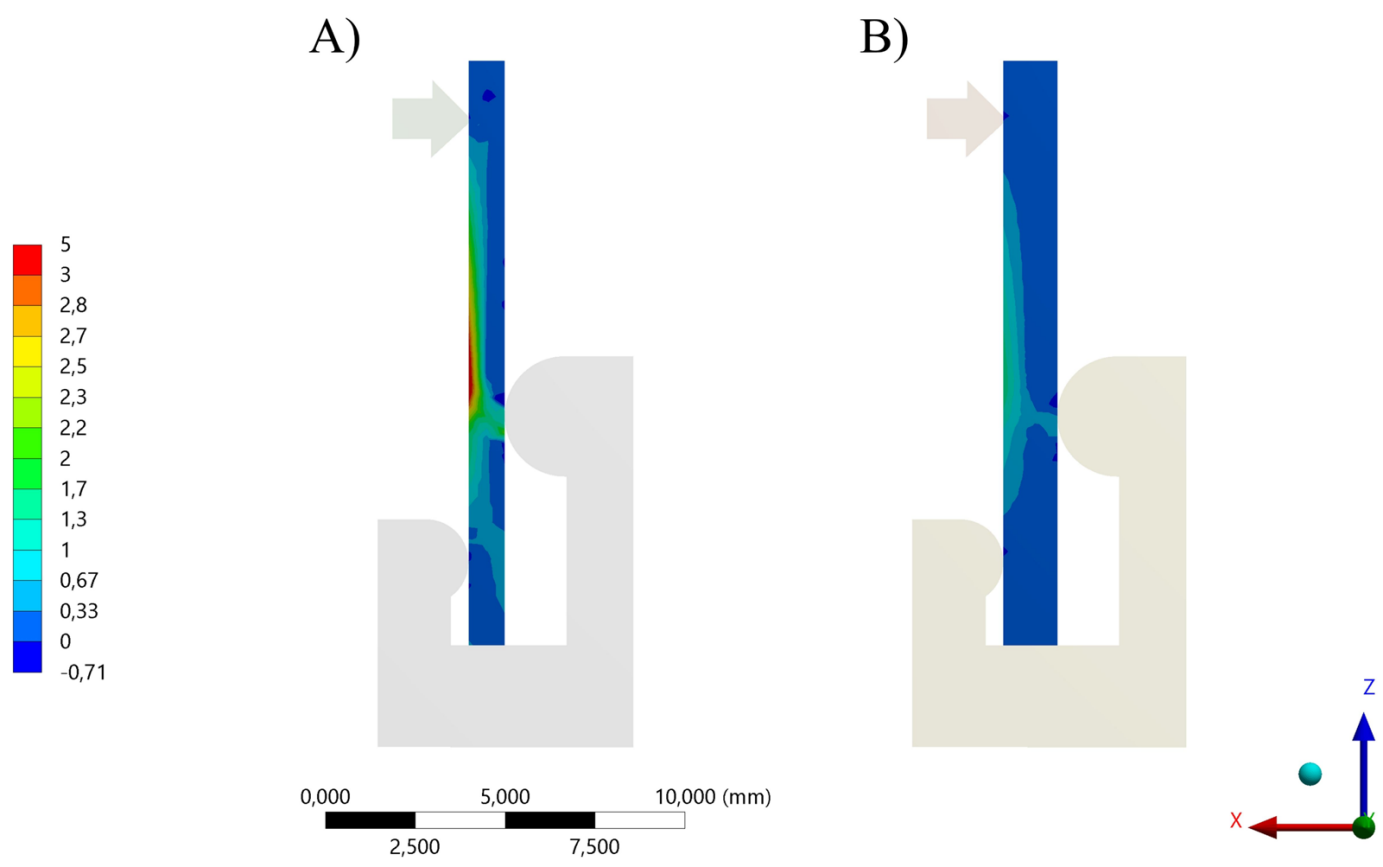
Maximum principal stress maps for the different thicknesses. (A) 1.0 mm thickness; (B) 1.5 mm thickness.

Games-Howell post hoc tests revealed that 3Y-TZP exhibited significantly superior impact strength compared to both 5Y-PSZ (p &lt; 0.001) and TZ (p &lt; 0.001), while TZ showed intermediate performance significantly higher than 5Y-PSZ (p = 0.035). Regarding thickness, 1.5 mm specimens generally demonstrated higher impact strength than 1.0 mm specimens. The significant three-way interaction indicated distinct response patterns across thickness levels. For 1.0 mm specimens, both conventional and speed sintering protocols yielded similar results within each zirconia type. In contrast, for 1.5 mm specimens, speed sintering differentially affected materials: it improved impact strength in 3Y-TZP, while reducing it in 5Y-PSZ, reflecting material-dependent mechanical responses. This differential response to sintering protocol as a function of both material type and thickness explains the significant three-way interaction. - Stress Calculation The finite element analysis simulated the conditions of the impact strength test for both thicknesses tested. As shown in Figure 3, the maximum principal stress occurred at the center of the sample.

3

**Figure 4:**
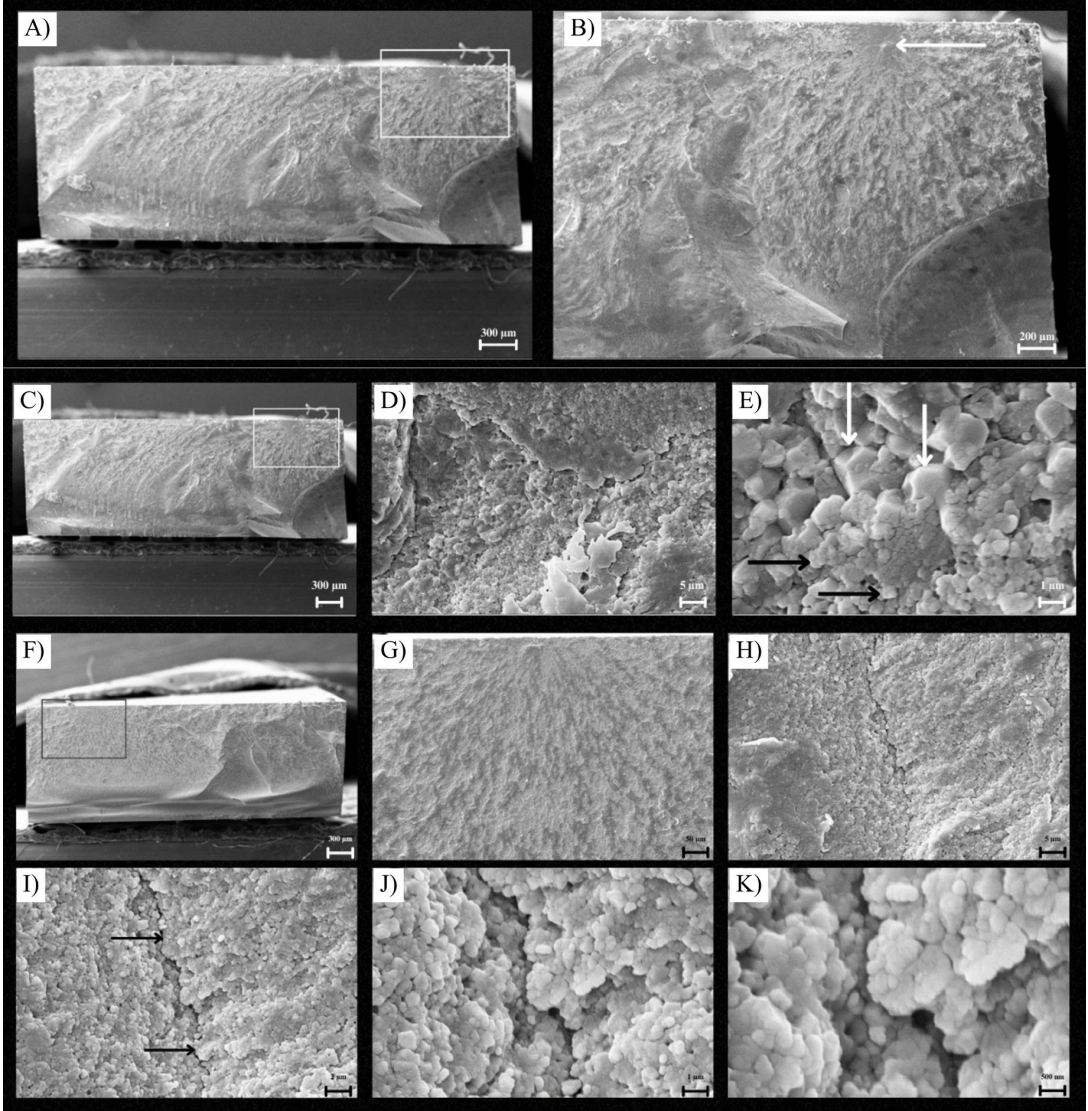
SEM analysis of graded zirconia: fractography, microstructure, and crack propagation. Fractographic analysis of the transition zone (TZ) is shown in (A–B). (A) Low-magnification overview (70×) of the fractured specimen, with the white rectangle indicating the region magnified in (B). (B) Higher magnification (150×) highlighting the fracture origin, identified as an internal flaw (white arrow). Microstructural characterization of the TZ is presented in (C–E). (C) Low-magnification overview (70×) showing the general morphology. (D) Intermediate magnification (5,000×) revealing grain distribution. (E) High magnification (25,000×) illustrating the coexistence of tetragonal (black arrows) and cubic grains (white arrows). Crack propagation features in the 5Y-PSZ layer (1.5 mm thickness, speed sintering) are shown in (F–K). (F) 70×, (G) 500×, (H) 5,000×, (I) 10,000×, (J) 25,000×, and (K) 50,000× magnifications. Intergranular crack propagation is observed (black arrows), with fracture origin indicated by the black rectangle.

The stress peak for the 1.0 mm thickness was 56.9 MPa, while the thicker model generated a maximum of 24.7 MPa. Fractographic analysis confirmed the origin of the fracture in the same region where the FEA indicated the highest stress concentration. - Fractography Fractography revealed similar fracture patterns regardless of the layer or sintering mode tested. The fracture origin was identified in almost all samples; however, in some cases, it was not possible to determine the exact origin of failure due to the presence of multiple secondary fractures and delamination. Figure 4 shows the fracture origin in a representative sample of the transition zone.

4

SEM evaluation of the TZ group also allowed the observation of the polycrystalline microstructure of zirconia, revealing a microstructural morphology consistent with the presence of cubic and tetragonal grains (Fig. 4), as well as intergranular fractures, as illustrated in Figure 4.

## Discussion

This study evaluated the impact strength of graded zirconia across its different zones (3Y-TZP, transition zone, 5Y-PSZ) under different thicknesses (1.0 and 1.5 mm) and sintering protocols (conventional and speed). The 3Y-TZP zone demonstrated the highest impact resistance across all conditions, followed by the transition zone, with the 5Y-PSZ showing the lowest values. The null hypothesis was rejected, confirming that impact strength varies across material zones, with interactions between sintering protocol and specimen thickness influencing mechanical performance. The significant three-way interaction between material zone, sintering protocol, and thickness (F2,168 = 5.080, p = 0.007) reveals complex interdependencies that govern graded zirconia performance under impact forces. Speed sintering protocols demonstrated differential effects across materials and thicknesses, with 5Y-PSZ at 1.5 mm thickness showing approximately 45% reduction in impact strength compared to conventional sintering. This finding could be attributed to the formation of transient thermal stresses during rapid heating, which is both thickness- and composition-dependent due to differential thermal expansion and contraction rates during these accelerated thermal cycles (16). According to Alshahrani et al. (2023), thicker specimens experience greater thermal gradients during speed sintering, leading to increased residual stress accumulation, particularly in cubic-rich regions that lack the stress accommodation mechanisms available in tetragonal phases. The higher standard deviations observed in thicker specimens support this interpretation, suggesting increased susceptibility to processing-induced defects and stress concentrations. Conversely, the 3Y-TZP layer showed enhanced performance under speed sintering at 1.5 mm thickness, indicating that transformation toughening mechanisms in tetragonal zirconia may benefit from rapid thermal processing under specific conditions. The superior impact strength of the 3Y-TZP layer (46.8 to 66.7 kJ/m²) compared to both transition and 5Y-PSZ layers stems from its microstructural advantage, specifically the predominantly tetragonal grain structure that enables stress-induced martensitic transformation. During mechanical loading, tetragonal grains undergo volumetric expansion of approximately 4-5%, creating compressive stress fields around crack tips that blunt crack propagation and enhance fracture resistance (2). These findings align with de Jager et al. (24), who reported 65.75 ± 6.07 kJ/m² for 2 mm thick 3Y-TZP specimens, validating the reproducibility of these mechanical characteristics across different testing configurations and reinforcing the reliability of transformation toughening as the primary strengthening mechanism in tetragonal zirconia. In marked contrast, the 5Y-PSZ layer's inferior performance (15.5 to 28.7 kJ/m²) reflects the inherent trade-off between mechanical strength and optical properties in cubic-dominated microstructures. The absence of transformation toughening in cubic grains, combined with their larger size and reduced packing density, creates a microstructure inherently more susceptible to crack initiation and propagation (4). This fundamental compromise between strength and translucency represents the core challenge that graded technology attempts to address through spatial optimization of properties. The transition zone's intermediate behavior (23.7 to 41.9 kJ/m²) provides evidence for gradient integration without creating mechanical weak points, despite containing mixed phases that could potentially generate stress concentrations due to thermal expansion mismatches (10 , 11). Our results confirm that this zone maintains structural integrity under impact conditions comparable to that of homogeneous materials, corroborating previous studies that demonstrated the mechanical integrity of this zone (6 , 8 , 22 , 26 - 30). Fractographic analysis revealed no evidence of preferential crack propagation along phase boundaries. Instead, intergranular fracture patterns were observed, suggesting that grain boundaries may represent weaker regions that could potentially affect mechanical performance. Our finite element analysis, conducted through representative modeling of the Dynastat impact resistance test, reveals that maximum stress concentration occurs at the central region of the specimen due to bending moment mechanics rather than at the distal point of load application. This phenomenon reflects the lever arm principle inherent in dynamic impact loading, where the sudden application of force at a distance from the fulcrum generates maximum tensile stress at the specimen's mid-span through flexural deformation (24 , 25). The consistent identification of fracture origins in regions of maximum calculated stress demonstrates that impact failure in this material follows predictable patterns, enabling rational design optimization. However, a limitation of this study is that material gradation was not modeled in the FEA, as the elastic modulus and Poisson's ratio of the constituent layers were considered similar, which is supported by the literature (4). Nevertheless, there remains scope for FEA models that explore how subtle gradations in elastic modulus and Poisson's ratio can influence stress dissipation and fracture resistance, particularly in the transition zone of these materials. In the present study, the correlation between finite element predictions and fractographic observations strengthens confidence in our mechanical interpretations and supports the development of predictive models for restoration design. The observed thickness effects directly inform restoration design protocols: while 1.0 mm thickness demonstrated acceptable performance across all zones and sintering modes, significant performance degradation of the 5Y-PSZ layer at 1.5 mm under speed sintering indicates that processing guidelines should include layer-specific thickness considerations. Our results suggest that speed sintering should be limited to restorations where the 5Y-PSZ layer thickness does not exceed 1.0 mm, while the 3Y-TZP and gradient zones maintained acceptable performance at increased thicknesses. This finding has particular relevance for restoration planning, since the degradation of individual layers could compromise the overall integrity of the multilayer system, especially in long-span prostheses where connector dimensions and anticipated loading patterns must be carefully considered during design to optimize layer distribution (7 , 26 , 27). Our results align well with recent investigations of graded zirconia systems while providing novel insights into impact behavior. The impact strength values obtained fall within ranges reported for conventional dental ceramics (23 , 24), indicating that graded zirconia maintains competitive mechanical performance while offering superior aesthetic properties. When compared to established dental materials reported by Jager et al. (2021), even the worst-performing condition in our study (5Y-PSZ, speed sintering, 1.5 mm - 15.5 ± 3.31 kJ/m²) demonstrated impact resistance comparable to or exceeding that of well-established ceramics such as lithium disilicate (15.72 kJ/m²) and zirconia-reinforced lithium silicate (14.41 kJ/m²), while substantially outperforming feldspathic ceramics (0.76 kJ/m²) and commercial composite materials (1.52-4.08 kJ/m²) (24). While the present study provides valuable data on the impact behavior of graded zirconias, the use of simplified bar geometries by layer cannot fully capture complex stress distributions in actual restoration configurations, warranting future investigations of impact resistance with anatomic specimens, including crowns, bridges, and complex prosthetic shapes. Additionally, the focus on abrupt impact loading, while relevant for trauma scenarios, does not address cyclic fatigue behavior under normal masticatory function. Although previous studies have investigated fatigue performance of this material considering different sintering protocols and design parameters, comprehensive mechanical characterization requires further exploration to fully understand all factors influencing mechanical properties, particularly considering the complex integration between different zirconia microstructures with varying crystallographic phases and yttria concentrations that occurs gradually throughout this material. Future research should encompass clinical trials and long-term case follow-ups, alongside long-term degradation studies incorporating environmental factors such as moisture, temperature cycling, and pH variations to provide robust scientific support for longevity predictions.

## Conclusions

This study demonstrates that impact resistance in graded zirconia systems is governed by complex interactions between material composition, sintering protocol, and specimen thickness. Key findings include: The 3Y-TZP layer exhibited superior impact strength across all testing conditions, confirming its suitability for high-stress applications. Conventional sintering proved optimal for 5Y-PSZ layers, with speed sintering causing significant impact strength reduction (~45%) in thicker specimens. The transition zone maintained intermediate impact resistance without creating structural weaknesses, confirming the robustness of graded systems. Thicker specimens showed increased susceptibility to processing-induced defects, emphasizing the importance of optimized manufacturing parameters. These findings provide a comprehensive impact resistance characterization of graded zirconia systems, offering evidence-based guidance for clinical material selection and processing protocols in high-impact clinical scenarios.

## Figures and Tables

**Table 1:** Descriptive statistics of impact strength obtained from each experimental group. Mean ± standard deviation (kJ/m²) according to material layer, sintering, and thickness (n=15 per group).

Material	Sintering	Impact Strength (kJ/m²)	
		1.0 mm	1.5 mm
3Y-TZP	Speed	56.5 ± 12.6	66.7 ± 23.1
	Conventional	49.0 ± 6.7	63.0 ± 19.5
TZ	Speed	23.8 ± 5.5	30.5 ± 15.8
	Conventional	26.8 ± 6.7	31.3 ± 7.97
5Y-PSZ	Speed	23.5 ± 4.2	15.5 ± 3.3
	Conventional	17.9 ± 5.9	35.3 ± 17.0

1

**Table 2:** Results of three-way ANOVA on the effects of material layer, sintering, and thickness on impact strength.

Source of Variation	Sum of Squares (SS)	Degrees of Freedom (df)	Mean Squares (MS)	F Statistic (F)	p-value (p)
Zirconia Type	44,991.4	2	22,495.7	145.232	< .001
Sintering	56.8	1	56.8	0.366	0.546
Thickness	2,500.3	1	2,500.3	16.142	< .001
Zirconia Type × Sintering	1,221.5	2	610.8	3.943	0.021
Zirconia Type × Thickness	487.4	2	243.7	1.573	0.210
Sintering × Thickness	910.3	1	910.3	5.877	0.016
Zirconia Type × Sintering × Thickness	1,573.6	2	786.8	5.080	0.007
Residual (Error)	26,022.3	168	154.9	—	—

Values in bold indicate statistically significant effects (p < 0.05). Interactions indicate that the effect of one factor depends on the level of another.
